# Rapid method for screening of both calcium and magnesium chelation with comparison of 21 known metal chelators

**DOI:** 10.1007/s00775-024-02078-6

**Published:** 2024-10-18

**Authors:** Lukáš Konečný, Zuzana Lomozová, Galina Karabanovich, Jaroslav Roh, Kateřina Vávrová, Přemysl Mladěnka

**Affiliations:** 1 Department of Pharmacology and Toxicology, Faculty of Pharmacy in Hradec Králové, Charles University, 50003 Hradec Králové, Czechia; 2 Department of Pharmacognosy and Pharmaceutical Botany, Faculty of Pharmacy in Hradec Králové, Charles University, 50003 Hradec Králové, Czechia; 3 Department of Organic and Bioorganic Chemistry, Faculty of Pharmacy in Hradec Králové, Charles University, 50003 Hradec Králové, Czechia

**Keywords:** Chelator, Depletion, Selectivity, Methodology, Platelet

## Abstract

Chelation is the rational treatment modality in metal overload conditions, but chelators are often non-selective and can, hence, cause an imbalance in the homeostasis of physiological metals including calcium and magnesium. The aim of this study was to develop an affordable, rapid but sensitive and precise method for determining the degree of chelation of calcium and magnesium ions and to employ this method for comparison on a panel of known metal chelators. Spectrophotometric method using *o*-cresolphthalein complexone (*o*-CC) was developed and its biological relevance was confirmed in human platelets by impedance aggregometry. The lowest detectable concentration of calcium and magnesium ions by *o-*CC was 2.5 μM and 2 μM, respectively. The indicator was stable for at least 110 days. Four and seven out of twenty-one chelators strongly chelated calcium and magnesium ions, respectively. Importantly, the chelation effect of clinically used chelators was not negligible. Structure–activity relationships for eight quinolin-8-ols showed improvements in chelation particularly in the cases of dihalogen substitution, and a negative linear relationship between pKa and magnesium chelation was observed. Calcium chelation led to inhibition of platelet aggregation in concentrations corresponding to the complex formation. A novel method for screening of efficacy and safety of calcium and magnesium ion chelation was developed and validated.

**Graphical abstract:**

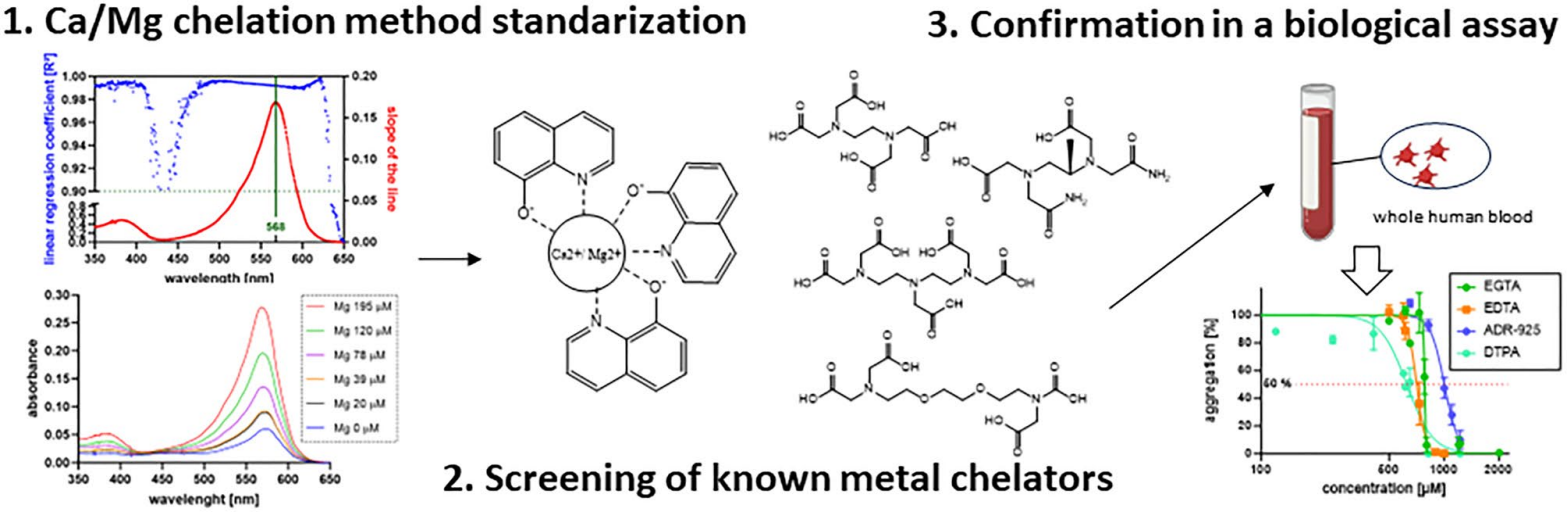

**Supplementary Information:**

The online version contains supplementary material available at 10.1007/s00775-024-02078-6.

## Introduction

Calcium and magnesium are metals essential for innumerable physiological processes ranging from being cofactors of enzymatic reactions to their involvement in complex processes including platelet aggregation and blood coagulation [1–3]. Deficit of these metals leads to disharmony in many biological pathways. Calcium depletion is rare but more dangerous than more frequently occurring hypomagnesemia [4]. In particular, the latter situation is much more common as the intake of magnesium from the diet is insufficient in about 60% of adults currently [3]. Overt deficiency in these metal ions can lead to serious pathological conditions such as extrapyramidal symptoms or heart failure or neuropsychiatric disorders such as apathy and delirium [5, 6].

There are several methods used for the detection of calcium and magnesium ions, and their chelation. These different methods include flow sequential injection, potentiometric sensor assay, atomic absorption spectrometry, flame atomic absorption, mass spectrometry, or ion selective electrode [7–11]. They have different advantages and disadvantages. The latter include high cost including requirement of specific expensive devices. Some of these methods cannot be used for our purpose, e.g., atomic absorption spectrometry measures the whole metal content, and hence it is unsuitable for assessment of metal chelation. There are also indicators forming colored complexes with both metals with subsequent spectrophotometric detection. Examples of such compounds are thymolphthalein, bromopyrogallol, arsenazo III or *o*-cresolphthalein complexone [7]. The later was selected in our experiments considering also the economic aspects, for instance, *o*-CC is four times cheaper than arsenazo III. In principle, UV–Vis spectrophotometric methods can be accessible worldwide, they are not specific apparatus demanding, easy to perform and when standardized also sufficiently precise. Our team has extensive experience with such methods as we have developed competitive screening approaches for the detection of chelation of iron, copper, zinc, and recently also cobalt [12, 13].

**Fig. 1:**
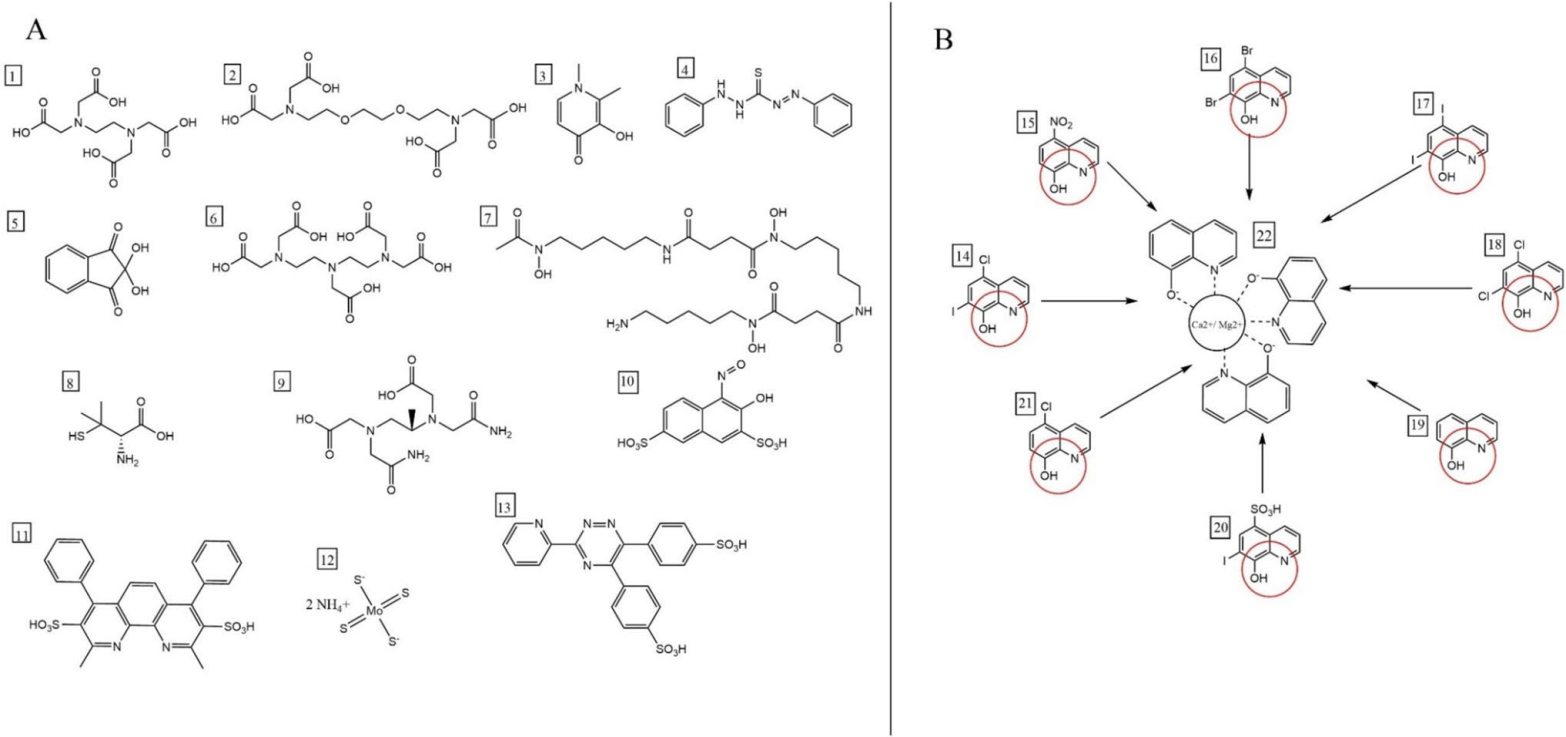
Examples of metal chelators used in this study. **A** Clinically and experimentally used metal chelators and **B** 8-hydroxyquinolines. (1) Ethylenediaminetetraacetic acid (EDTA), (2) ethyleneglycol-bis(2-aminoethylether)-*N,N,N′,N′*-tetraacetic acid (EGTA), (3) deferiprone, (4) dithizone, (5) ninhydrin, (6) diethylenetriaminepentaacetic acid (DTPA), (7) deferoxamine, (8) D-penicillamine, (9) ADR-925, (10) 1-nitroso-2-naphtol-3,6-disulfonic acid disodium salt hydrate (NNDSA), (11) bathocuproinedisulfonic acid disodium salt hydrate (BCS), (12) ammonium tetrathiomolybdate (ATTM), (13) ferrozine, (14) clioquinol, (15) nitroxoline, (16) broxyquinoline, (17) iodoquinol, (18) chloroxine, (19) 8-hydroxyquinoline, (20) chiniofon, (21) cloxiquine, (22) a possible complex of 8-hydroxyquinoline with calcium or magnesium ions in a stoichiometric ratio, 3:1. Chelation site is circled in red

There are many clinically used or experimentally tested chelators (Fig. 1). They are used mostly for metal overload conditions either iatrogenic or genetically based such as secondary hemochromatosis or Wilson’s disease, respectively [14]. In principle, chelators should be as selective as possible toward the metal of interest. This is, however, almost impossible but there is apparently the difference in affinity toward different metals. Affinity toward different metals can be measured by assessing stability constants [15] or their chelation activity can be assessed by other methods, e.g., by mentioned competitive screening methods [12, 13]. This study follows the later approach toward calcium and magnesium. Finding a chelator with higher activity toward calcium or magnesium might be clinically important from two aspects: (a) developing calcium and magnesium chelators as alternative treatment option for rare intoxication with one of these metals, (b) providing information that such chelator can cause or worsen depletion of these metals with consequent side effects. There are relevant examples for both metals; e.g., EDTA therapy causes calcium depletion when exposed in a long-term basis [16] and there are proposed unexpected side effects associated with possible lowering of magnesium levels by glucocorticoids [17].

To obtain a deeper insight in the current situation with calcium and magnesium chelation, a novel method for detection of calcium and magnesium chelation was standardized and a relatively large panel of known clinically or experimentally used metal chelators including eight structural derivatives of 8-hydroxyquinolines (Fig. 1) for assessing structure–activity relationships was tested and the biological relevance of our finding was confirmed in ex vivo experiments in whole human blood.

## Materials and methods

### Chemicals

#### Metals and indicator

Calcium chloride dihydrate (CaCl_2_·2H_2_O), magnesium sulfate heptahydrate (MgSO_4_·7H_2_O), *o*-cresolphthalein complexone (*o*-CC) were purchased from Sigma-Aldrich (Germany).

#### Chelating agents: quinolines

Broxyquinoline (5,7-dibromoquinolin-8-ol), clioquinol (5-chloro-7-iodoquinolin-8-ol), chloroxine (5,7-dichloroquinolin-8-ol), cloxiquine (5-chloroquinolin-8-ol), 8-hydroxyquinoline (quinoline-8-ol), nitroxoline (5-nitroquinolin-8-ol), chiniofon (8-hydroxy-7-iodoquinoline-5-sulphonic acid) were purchased from Sigma-Aldrich, whereas iodoquinol (5,7-diiodoquinolin-8-ol) was purchased from Toronto Research Chemicals (Canada).

#### Chelating agents: others

Ammonium tetrathiomolybdate (ATTM), bathocuproinedisulfonic acid disodium salt hydrate (BCS), disodium ethylenediaminetetraacetate dihydrate (EDTA), dithizone (1,5-diphenylthiocarbazone), d-penicillamine, ethyleneglycol-bis(2-aminoethylether)-*N,N,N′,N′*-tetraacetic acid (EGTA), ferrozine, ninhydrin were purchased from Sigma-Aldrich. 1-Nitroso-2-naphtol-3,6-disulfonic acid disodium salt hydrate (NNDSA) was purchased from Acros organics (USA); deferiprone (3-hydroxy-1,2-dimethyl-4(1H)-pyridone) from a gift from Apotex (Canada); pentasodium diethylenetriaminepentaacetate (DTPA) was bought from ThermoFisher (USA); and deferoxamine mesylate from Novartis (Switzerland).

ADR-925 was prepared in house as described previously. Briefly, dexrazoxane (1 g, synthesized according to the known procedure [18]) was stirred in 0.5 M aqueous NaOH (74.6 mL) at room temperature for 24 h. Upon completion, the solution was acidified to pH 5–6 with Amberlyst 15, filtered, and the filtrate was evaporated to dryness under vacuum. The resulting solid was further dried over P_2_O_5_ under vacuum in a desiccator for 7 days [19].

#### Solvents and other chemicals

Methanol (≥ 99.9%) was from Fisher Chemical (UK), while dimethyl sulfoxide (DMSO) was from Penta (Czech Republic). Ultrapure water (Milli-QRG, Merck Millipore, Massachusetts, USA) was used throughout this study. Sodium acetate, acetic acid, 4-(2-hydroxyethyl)-1-piperazineethanesulfonic acid (HEPES), HEPES sodium salt were purchased from Sigma-Aldrich and used for buffers. Acetate buffers (15 mM of sodium acetate and 27.3 or 2.7 mM of acetic acid) were used for the pH values of 5.5 and 4.5, respectively, whereas HEPES buffers (15 mM of sodium HEPES and 71.7 and 14.3 mM of HEPES, respectively) were employed for pH values of 6.8 and 7.5.

### Methods

#### Detection of UV–Vis absorption maxima and optimal wavelength(s) for absorption

The absorption spectra of *o*-CC dissolved in buffer of pH 7.5 at a final concentration 450 µM, and its mixtures with aqueous solutions of calcium and magnesium ions, were measured in the range of 200–800 nm every 0.5 nm (final concentrations of both ions were 450 µM). As a blank, the buffer with ultrapure water was used. Based on results of these initial measurements, following experiments were performed with absorbance measurement in the range of 350–650 nm. To find the optimal wavelength(s), different concentrations of both ions were mixed (in final concentrations of 20–195 µM) with *o*-CC (450 µM). Absorption spectra were measured by the spectrophotometer Helios Gamma (Spectronic Unicam, The United Kingdom). The same experiments were performed without the buffer with *o*-CC dissolved in methanol.

#### Detection of the sensitivity (the lowest detectable concentration)

A similar approach as with optimal wavelength was also applied for the assessment of the lowest detectable concentration of both ions. Calcium and magnesium ions in final concentrations of 0–5 µM were mixed with *o*-CC (again 450 µM), and absorption at optimal wavelengths based on the previous step was measured by the spectrophotometer.

#### Transforming the method to 96-well microplates

In the next step, the method was transformed to microplates to be easily usable for future screening of chelation. Also, the impact of lower pH was assessed. Briefly, 50 µL of ions (calcium or magnesium) in increasing concentrations were mixed with 150 µL of buffer (4.5–7.5) and 50 µL of water as the solvent for several tested chelators. After 1 min of mixing, 50 µL of 4 mM *o*-CC was added. Absorbance was measured immediately and after 5 min by the microplate spectrophotometer Hidex Sense Multimodal Microplate Reader (Hidex, Turku, Finland) at optimal wavelengths established in this research paper.

#### Verification of the stability of absorbance and the reagents

The stability of the reagents was verified by measuring the spectra every 7–14 days up to 147 days. Comparison was made with 3 mM stock solutions of both ions and *o*-CC solutions prepared on the day 1 stored in fridge (2–8 °C) with freshly prepared new *o*-CC solutions at day of the measurement. Measurements were performed in 96-well microplates where 150 µL of buffer 7.5 was mixed with 50 µL of mentioned calcium or magnesium ions and 50 µL of the *o*-CC solution. Absorbance was measured immediately and after 5 min at wavelengths selected for both ions.

#### Testing the methodology on various chelators

Calcium or magnesium ions at final concentration of 500 µM (50 µL) were mixed with buffer 7.5 (150 µL) in a microplate. After that, 50 µL of a tested agent or its solvent (water or DMSO) in different concentration was added. After 1 min of agitating, *o*-CC (50 µL) dissolved in buffer 7.5 was added (in a final concentration of 670 µM) and the absorbance was measured immediately and after 5 min at selected optimal wavelengths.

### Platelet aggregation experiment

#### Blood collection

Two healthy volunteers with mean age of 27 participated in this study. Medicines known to affect platelet aggregation (e.g., non-steroidal anti-inflammatory drugs) or alcohol were not allowed 24 h prior to blood collection. Blood samples were collected by venepuncture into plastic disposable syringes containing either heparin sodium (17 IU/mL). The collections were performed in the morning at 8–9 a.m. on a fasting gut. The project was approved by Human Research Ethics Committees of the Faculty of Pharmacy, Charles University No. UKFaF/176666/2021-2 from May 13, 2021. Every donor signed the informed consent.

#### Assessment of platelet aggregation

Shortly, 300 µL of whole blood was first diluted with the same volume of preheated saline solution (37 °C). 5 µL of EGTA, EDTA, ADR-925 or DTPA (final concentration in range 80–2000 µM) or saline as a negative control was added and incubated for 3 min at 37 °C. Platelet aggregation was then induced with arachidonic acid (Roche, Switzerland) at the lowest concentration causing maximal response based on our previous calibration method [20] and monitored for 6 min using the impedance aggregometer Multiplate (Roche, Switzerland). The aggregation response was quantified using the AUC (area under the curve).

### Mathematical calculation of the chelation and statistical analysis

Experiments were performed mostly in triplicates. Results are shown as mean ± SD or graphs with 95% confidence intervals. GraphPad Prism version 10.0.2 (San Diego, California, USA) was used for all data analyses. Parametric sample *t *test or unpaired sample *t *test was used in comparison of two dependent or independent sample, respectively.

Chelation of calcium and magnesium ions was calculated using following equation:

$$\left[ \% \right] \, = \left( {1 - \frac{{A_{x} - \overline{{A_{{\text{N}}} }} }}{{A_{{\text{P}}} - \overline{{A_{{\text{N}}} }} }}} \right) \times 100,$$

*A*_*x*_—the sample absorbance (chelating agent + metal ion + *o*-CC). *A*_N_—absorption of negative control (*o*-CC). *A*_*P*_—absorption of positive control (metal ion + *o*-CC).

Expected chelation in per cent (Y) at the ratio 1:1 was calculated form the equation:

$$Y = \frac{100}{{(1 + 10^{{\left( {\left( {{\text{LogEC50}}} \right) \times k} \right))}} }},$$

where *k* is the slope of the chelation curve and EC_50_ is the concentration needed for chelate 50% of the metal ion in the solution.

Aggregation was calculated using the following equation:

$${\text{aggregation }}\left[ \% \right] \, = \left( {\frac{{\text{AUC of the tested compound}}}{{\text{AUC of blank}}}} \right) \times 100.$$

## Results

### Development and standardization of the method

#### UV–Vis spectrum of o-CC with calcium and magnesium ions and optimal wavelength detection

**Fig. 2:**
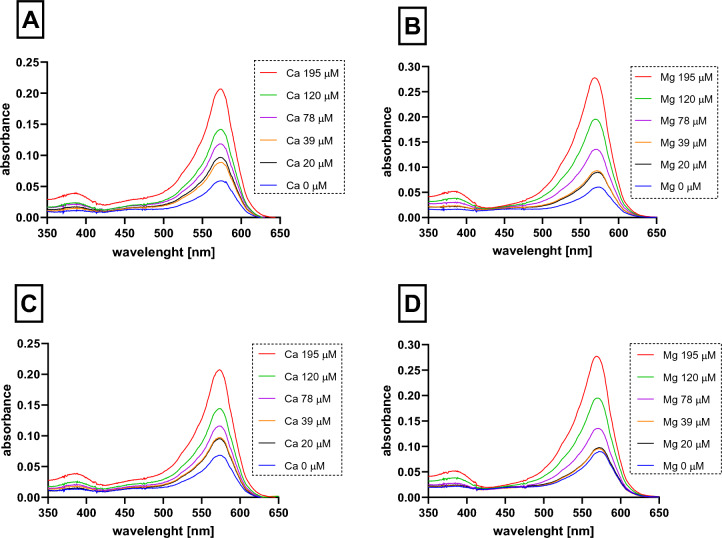
The spectra of indicator *o*-cresolphthalein complexone (*o*-CC) and its complex with calcium and magnesium ions at pH 7.5. **A** The complex of *o*-CC with calcium ions. **B** The complex of *o*-CC with magnesium ions. **C** The complex of *o*-CC with calcium ions after 15 min. **D** The complex of *o*-CC with magnesium ions after 15 min. The final concentrations of both ions ranged from 0 to 195 µM, while that of *o*-CC was 450 µM

First, the spectra of the indicator and its complex with calcium and magnesium ions at pH 7.5 (Fig. 2) and in non-buffered conditions in methanol (Supplementary Fig. S1) were measured. There was one clear peak at 574 ± 1 nm and one almost negligible peak at 388 ± 1 nm in the UV–Vis spectra of the indicator *o*-CC at pH 7.5. Addition of calcium ions resulted in a slight hypsochromic shift of the major peak to 573 ± 0 nm and intensification of the minor peak at 388 ± 0 (Fig. 2A). A similar situation was observed with Mg; addition of magnesium ions resulted as well in a slight hypsochromic shift of the major peak to 568 ± 0 nm and intensification of the minor peak at 386 ± 2 (Fig. 2B). The situation was analogous after 15 min (Fig. 2 CD) and when methanol was used instead of the buffer pH 7.5 (Supplementary Figure S1), but the absorption was lower in the latter case.

**Fig. 3:**
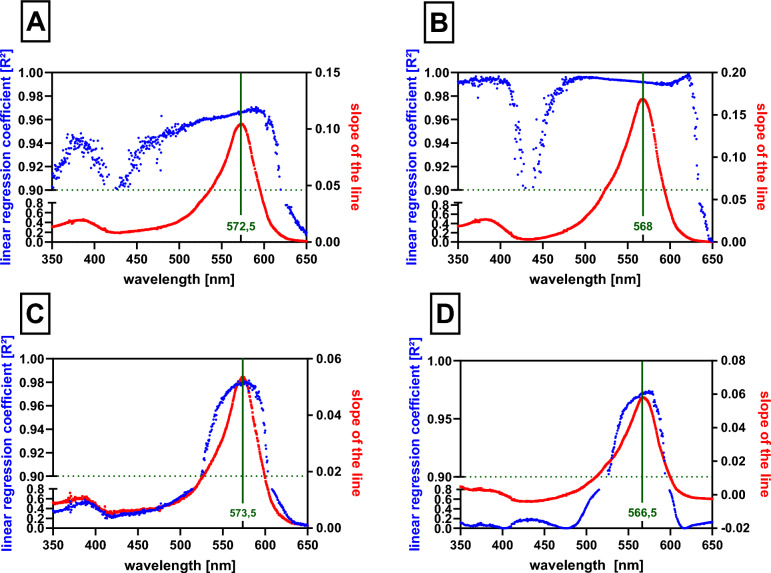
Detection of optimal wavelength for determination of calcium and magnesium ions using *o*-cresolphthalein complexone (*o*-CC). **A** The complex of *o*-CC with calcium ions in buffer 7.5. **B** The complex of *o*-CC with magnesium ions in buffer 7.5. **C** The complex of *o*-CC with calcium ions in methanol. **D** The complex of *o*-CC with magnesium ions in methanol. Left *y* axis is the measure of linearity (in blue), while the right axis is the gauge of sensitivity (in red). The optimal wavelengths based on these criteria are highlighted in green. The final concentrations of both ions ranged from 0 to 195 µM, while that of *o*-CC was 450 µM

As the spectra of the pure indicator and its complexes with tested metals were essentially similar, we decided to perform a detailed analysis to find optimal wavelenght(s) for purposes of our study. The analysis was based on two factors—linearity between absorption and concentration of metals, and sensitivity, i.e., steepness of the same relationship between absorption and concentration. These factors were expressed as the coefficient of linear regression (*R*^2^) and slope (*k*) of this linear relation, respectively. In experiments with buffer of pH 7.5, a sufficient linearity (*R*^2^ > 0.9) was observed for both calcium and magnesium at wavelengths ranging from 350 to 618 nm and 350 to 632.5 nm, respectively. Taking in the consideration the sensitivity, the optimal wavelength for detection was 572.5 nm (*R*^2^ = 0.965) and 568 nm (*R*^2^ = 0.991, Fig. 3A, B) for calcium and magnesium, respectively. In case of methanol (Fig. 3C, D), the sensitivity was clearly lower as can be observed from differences of slopes at both conditions. The highest sensitivity overlapped with highest linear regression values, and hence suitable wavelengths for detection of calcium were from 524.5 to 603.5 nm and of magnesium from 527 to 593.5 nm under these conditions (Fig. 3C, D). The optimal wavelengths were 573.5 and 566.5 nm for calcium and magnesium, respectively.

#### The limit of detection of both ions

**Fig. 4:**
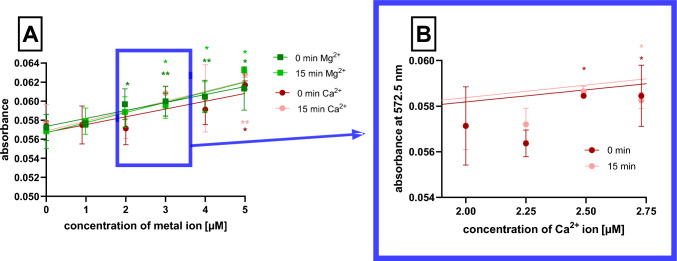
Detection of the sensitivity. **A** Dependence of absorbance on the concentration of calcium ions at 572.5 nm and magnesium ions at 568 nm. **B** A detail for calcium ions at 572.5 nm. *p < 0.05; **p < 0.01 vs. negative blank without ions

Using the approach described above, the lowest concentration of calcium ions that was detectable with significant difference from the negative blank was 2.5 µM (Fig. 4). For magnesium ions, the sensitivity of the method was even better as it was possible to detect 2 µM concentration. The detection potential somewhat decreased with time as after 15 min, the lowest concentration increased to 2.7 µM for calcium ions and to 3 µM for magnesium ions.

#### Verification of the stability of absorbance and the reagents

The stability of the *o*-CC indicator measured by its ability to form complex with calcium and magnesium ions was verified in a long-term period. The same complex with magnesium and calcium as with fresh solution was maintained for at least 110 days when the solution was kept at 4 °C (Supplementary Fig. S2). As the measurement was performed for 150 days and, hence, during different seasons of the year, and temperature of measurement impacts absorbance, the temperature in the laboratory and in the spectrophotometer was monitored during measurement together with absorbance to eliminate possible confounders. The temperature ranged from 22 to 28 °C, and from 23 to 28 °C, in the laboratory and in the instrument, respectively. However, there was no correlation between absorbance and temperature of the measurement showing that the impact of temperature was not a confounding factor (Supplementary Fig. S3 and Supplementary Table S1).

#### Effect of pH

**Fig. 5:**
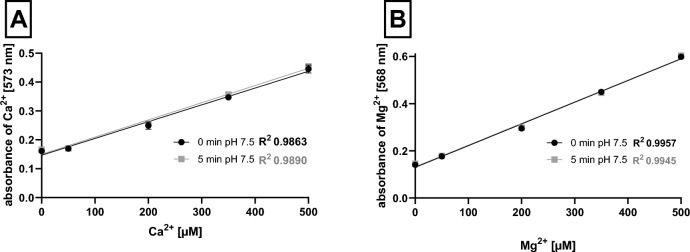
Calibration lines of both ions at pH 7.5 in microplate assay. **A** Calcium ions. **B** Magnesium ions. Data are for absorbance measured immediately and after 5 min. Data for other pH conditions are shown in Supplementary Fig. S3

In the next step, the method was successfully transformed from cuvettes to microplates as was confirmed by linearity between absorption and calcium/magnesium concentrations at pH 7.5 (Fig. 5). However, with decreasing pH, the sensitivity of the method markedly dropped (at pH 6.8) or was lost (at pH 5.5 and 4.5, Supplementary Fig. S4). The method could be, hence, employed solely at neutral conditions.

#### Validation of the methodology on various chelators and comparison of their effect

After the successful standardization of the protocol, the method was validated on 21 different known chelators which were composed from 8 structurally related quinolin-8-ols and 13 chemically variable clinically and experimentally used chelating agents (Fig. 1).

**Fig. 6:**
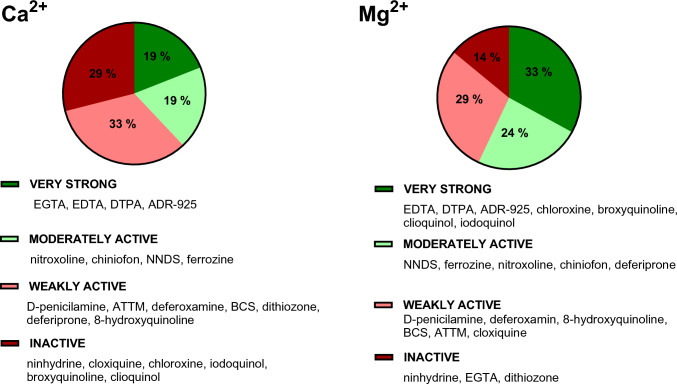
Potency of the tested chelators toward both ions. Twenty-one substances were included in the analysis (100%)

**Fig. 7:**
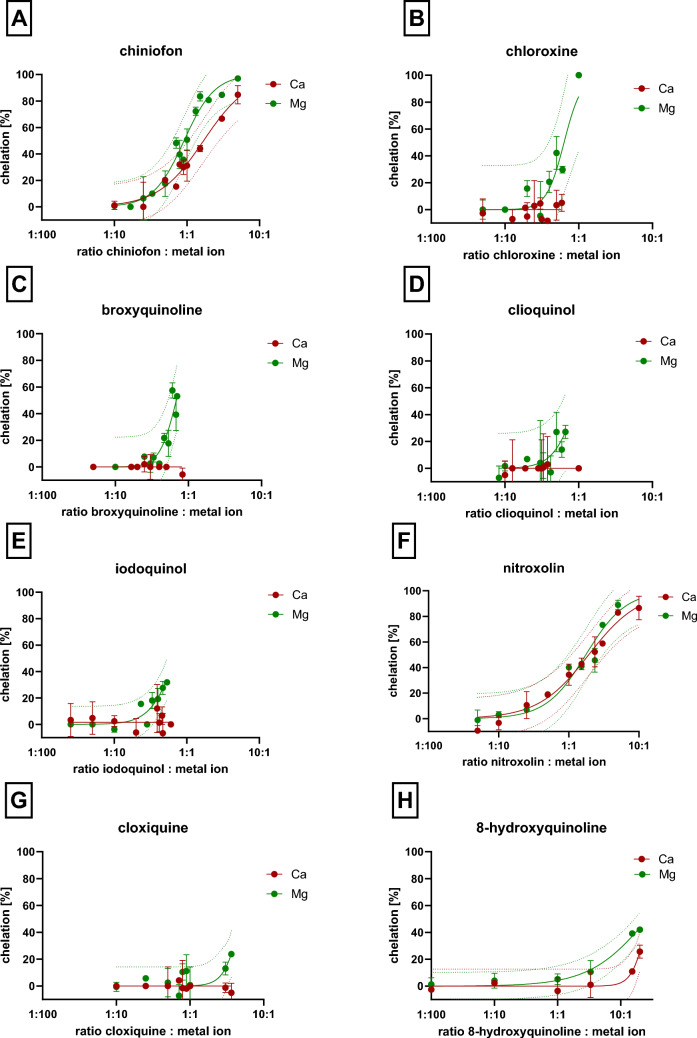
Comparison of the chelating activity of eight quinolines for calcium and magnesium ions. The chelating activity of quinolines with calcium ions was measured at 572 nm, while that of magnesium ions at 568 nm. Results are presented as mean with 95% confidence interval. The final concentration of either ion was 0.5 mM. The final concentrations of tested compounds were in the range 270 µM to 25 mM depending on their chelating activity and solubility

**Fig. 8:**
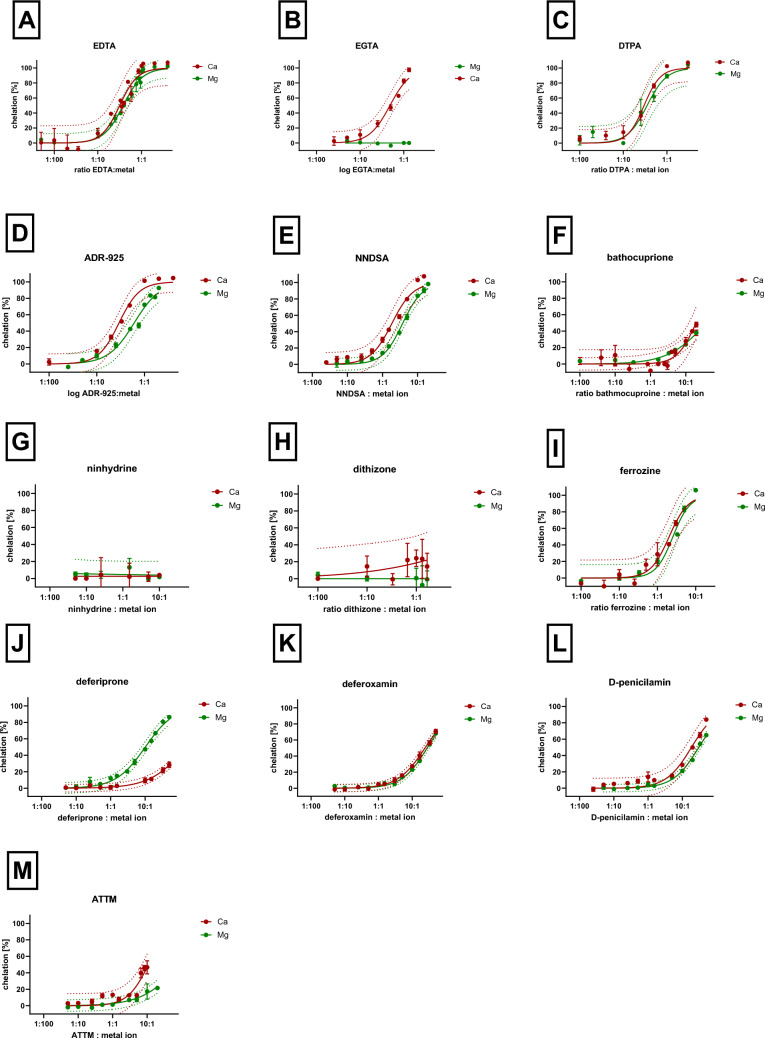
Comparison of the chelating activity of other 13 metal chelating substances for calcium and magnesium ions. The chelating activity of all various substances with calcium ions was measured at 572 nm, while that of magnesium at 568 nm. Results are presented as mean with 95% confidence interval. The final concentrations of both ions were 0.5 mM. The final concentrations of tested compounds were in the range 270 µM to 25 mM depending on their chelating activity and solubility

The chelators were divided into four categories: very strong magnesium and calcium chelators, moderately active, weak chelators, and inactive chelators (Fig. 6). In the case of very strong chelators, it was possible to assess the stoichiometry of the complex from our measurements. There were eight compounds in this category, but it needs to be emphasized that some compounds chelated calcium or magnesium more strongly. In case of calcium, four chelators can be considered strong: EDTA, EGTA, ADR-925, and DTPA. All formed complexes with calcium in the stoichiometric ratio of 1:1. In case of magnesium, EDTA, ADR-925, and DTPA had the same potency as they formed 1:1 complexes, while EGTA was inactive. In addition, there were five quinolines (chloroxine, broxyquinoline, clioquinol, iodoquinol and chiniofon) which in contrast to calcium, strongly chelated magnesium and seemed to form complexes 1:1 or 3:1. Unfortunately, some of them are by itself or in the form of their metal complexes weakly soluble under our experimental conditions, so full chelation curves could not be always constructed. Moderately active chelators were able to chelate completely magnesium and/or calcium ions at the ratio 10:1 but their stoichiometry cannot be drawn from our method. Weakly active chelators achieved at least some significant chelation at a ratio of 10:1, chelator to metal. Inactive chelators for both calcium and magnesium were solely ninhydrin and dithizone, whereas the abovementioned EGTA was inactive for magnesium (Figs. 7 and 8).

In most cases, the chelator chelated both calcium and magnesium ions with the same efficacy. There were few exceptions, in addition to the abovementioned EGTA, also ADR-925, TTM and NNDSA chelated significantly more strongly calcium ions when compared to magnesium, whereas the opposite was true for deferiprone. There seems to be also differences in five quinolines, which appeared to chelate only magnesium, namely chloroxine, broxyquinoline, clioquinol, cloxiquine, and iodoquinol. These data are, however, limited to low ratios due to the mentioned solubility issues and hence for testing higher ratios, other method will be needed.

**Fig. 9:**
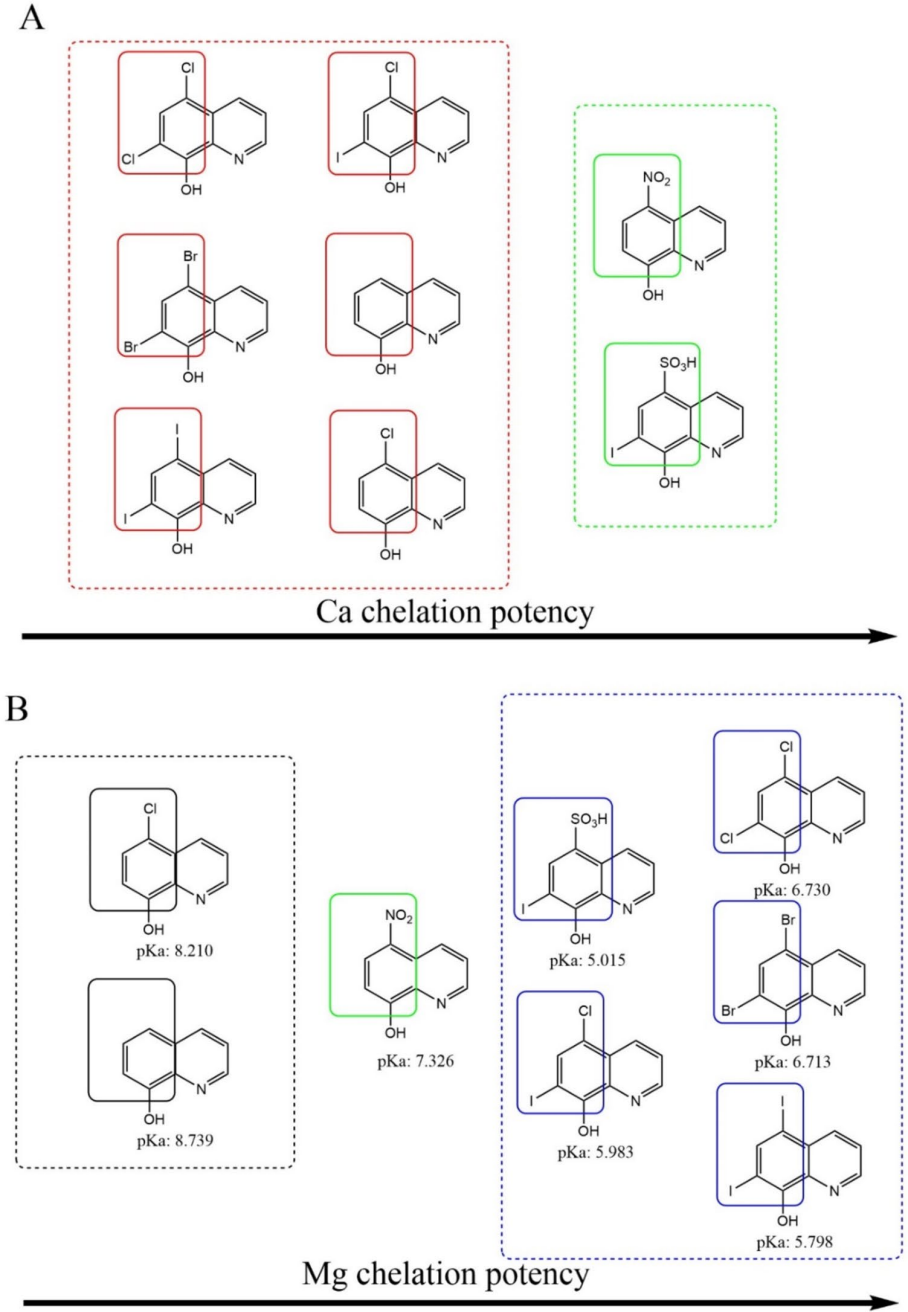
Summary of chelation potency of tested quinolines. **A** Potency of calcium chelation. Green—moderately active (in a chelator/metal ratio of 10:1), red—no effect (in the tested concentrations). **B** Potency of magnesium chelation. Blue—the most potent compounds, green—moderately active compounds, black—mild effect. The direction of arrow points to a more potent chelator. There were no significant differences among compounds with the same color. pKa values were calculated using ChemDraw 22.2.0

**Fig. 10:**
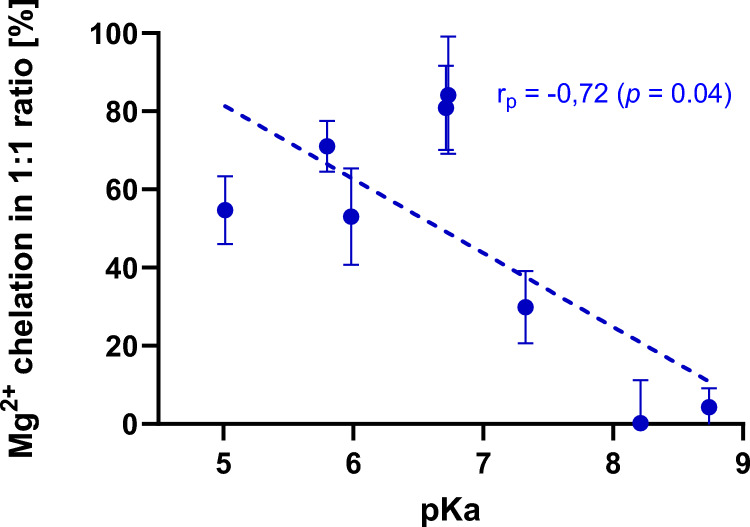
The negative linear relationship between acidity (calculated values of p*K*_a_) of hydroxyl in the chelating site of 8-hydroxyquinolines and chelation effect. Percent of chelation was calculated from the chelation curve (see the chapter on mathematical calculation)

With the same limitations, a structure–activity relationships for 8-hydroxyquinoline and its 7 close derivatives were analyzed by comparing 95% confidence intervals of their chelation curves (an example is shown in Supplementary Fig. S5). It was found that the most effective magnesium chelating quinolines were disubstituted at the 5 and 7 positions of the phenolic part of the molecule with the halogen or sulfonic acid. The type of halogen had no impact. In contrast, monosubstitution with only one chlorine at position 7 was associated with lower chelation effect. Contrarily, a nitro group in the same position led to a compound able to chelate both magnesium and calcium with moderate potency. This pattern was, however, less active in magnesium chelation when compared to dihalogen and chiniofon (Fig. 9). As substituents are known to impact the acidity of the hydroxyl group in the metal binding moiety, the relationship between pKa and chelation effect was analyzed. In the case of magnesium, lower pKa meant significantly higher chelation effect (Fig. 10).

### Confirmation of calcium chelation in whole human blood

**Fig. 11:**
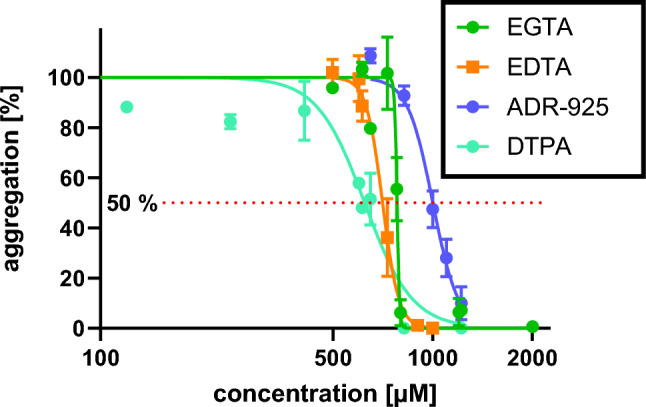
Verification of the most effective calcium chelators in a biological sample. Inhibition of platelet aggregation triggered by arachidonic acid (final concentration ranged from 70 to 120 µM based on calibration). Results are presented as mean ± SD

The most potent calcium ion chelators soluble in water, a solvent most compatible with biological testing, were evaluated for biological relevance of our findings. We employed aggregation of human platelets as this process is crucially dependent on calcium, and calcium concentration in human blood is stable around 2 mM under physiological conditions [21]. Based on our conditions, where the blood was diluted by the same volume of saline, and our results, where these compounds formed complexes 1:1, full inhibition of platelet aggregation should occur at a concentration of 1 mM of a respective chelator, and this was indeed observed (Fig. 11). The inhibitory effect of ADR-925 was slightly inferior but our experiments were designed to confirm our in vitro effect but not to detect difference as the precise concentration of calcium in blood was not measured.

## Discussion

Increasing industrial activity with industrial wastewater or mining is associated with metal exposure in populations together with possible negative consequences on humans [22]. For this reason, chelators of heavy metals, in particular, are increasingly required. In addition, there are genetic or iatrogenic metal overload conditions encompassing Wilson’s disease and hemochromatosis requiring the pharmacological treatment. The consumption of metal chelators is already high as can be assessed from the frequency of metal ion diseases since there are, for example, currently 16 million Americans suffering from some degree of iron overload. The abovementioned Wilson’s disease is quite a common metabolic disease in the world with prevalence 1:30,000 so potentially 260,000 people are taking a chelating agent (e.g., tested d-penicillamine) [23–25].

The therapy using chelating agents should be selective and the affinity of these chemicals has to be much higher for toxic metals than for biogenic microelements [26, 27]. It is, however, also known that chelators form frequently non-selectively complexes with other microelements such as calcium or magnesium ions. This can logically be associated with side effects [28, 29]. Similarly, calcium and magnesium share many common physicochemical aspects, including the same oxidation state of stable ions and being both hard acids. Hence, preparation of selective chelators toward one of these metals is not an easy task as was already repeatedly reported [30, 31] and also documented in this study. From 13 included non-quinoline chelators, 2 did not significantly chelated none of these metal ions in the tested concentrations (ninhydrin, dithizone), while 10 chelated both and solely EGTA was selective for calcium. From those ten compounds which chelated both, significant differences between chelation potencies toward calcium or magnesium were observed in four cases but two of them were very marginal (ADR-925, NNDSA). The clinical relevance of observed chelation effects is important mainly for deferoxamine, deferiprone, D-penicillamine, ATTM, and ADR-925. The first two compounds are used for iron overload, the third and fourth in Wilson´s disease, and ADR-925, is the active metabolite of dexrazoxane, used for prevention against anthracycline cardiotoxicity. As far as we know, there are only few reported data on interaction of these chelators with calcium and magnesium, which is quite interesting as all of them were found to have not negligible chelation effects toward both metals (Fig. 8). It is known from literature that deferoxamine is able to chelate and make complexes with 20 metal ions including bivalent ions [32] and ADR-925 can bind calcium and magnesium with almost same potency as iron or copper [33]. More interestingly, there is a study reporting that penicillamine does not form complexes with calcium or magnesium ions [34]. We have, however, observed that this compound can chelate both ions in ratios of 10:1 (metal:ions) and higher (Fig. 8). This might be an advantage of our approach, that it can identify also relatively weak chelators. Regardless, this also confirms the hypothesis of non-selectivity of chelators in general.

The remaining eight chelators from the class of 8-hydroxyquinolines represent known chelators whose multiple chelation effects with potential application in various diseases ranging from neurogenerative disorders, to cancer and microbial infection [35] are already known. On the other hand, non-selectivity toward various metals can lead to, as mentioned above, a higher risk of side effects and they are not currently approved for any systemic indication. These compounds are bidentate chelators which form stable complexes with metal ions due to a short distance between chelating atoms, heterocyclic nitrogen, and hydroxyl oxygen (Fig. 1B). The stability of these complexes can be decreased in acidic pH [13, 36, 37]. This general ability is known also in other classes of chelators, but introduction of halogen or other appropriate substituents can, however, lead to stable complexes of 8-hydroxyquinolines also in acidic pH as was observed for copper [13]. In this study, we were not able to test the chelation also under different pH conditions due to inability of the indicator to bind calcium and magnesium under these conditions. On the other hand, as 8-hydroxyquinolines included in this study form a group of structurally close derivatives, and therefore some structural conclusions can be drawn toward chelation of calcium and magnesium under neutral conditions. These data extend limited previous results in this area. It was reported that clioquinol is able to chelate calcium and magnesium although its affinity toward these metals was lower when compared to copper and zinc [38]. Stability constants for some derivates of 8-hydroxyquinoline with calcium and magnesium were also reported but our tested quinolines were not included [39]. Starting from 8-hydroxyquinoline, its substitution with chlorine in position 5 did not improve the chelation effect neither toward Ca nor Mg. Contrarily, introduction of a second halogen always improved the chelation effect toward Mg, but it decreased the solubility in water and hence prevented a similar analysis in case of calcium to which 8-hydroxyquinolines have apparently lower affinity. Interestingly, introduction of a nitro group at position 5 improved the Ca/Mg chelation effect comparing to the parent compound, 8-hydroxyquinoline. The improving effect was lower when compared to dihalogenated quinolines in relation to magnesium chelation. Introduction of a sulfonic group improved the water solubility but also lead in combination with the iodine atom at position 7 to improvement of Mg and Ca chelation effects. The first logical explanation of these differences is that the electron withdrawing effects of the substituents render the chelating hydroxy moiety more acidic and, hence, more ionized and available for chelation at around neutral pH. This explanation fitted to magnesium chelation results as was confirmed by the negative linear relationship between calculated values of pKa and chelation effect (Fig. 10), but the chelation pattern toward calcium is more complex. Apparently other previously described factors including denticity, different size of the metal ion, and solvation may contribute to the final picture. In fact, chelators with lower denticity (8-hydroxyquinolines are solely bidentate ligands as mentioned above) prefer magnesium over calcium [31]. This appears to agree with our data. Another aspect might be the stability of the formed complexes, as they compete with *o*-CC for the metal. It is possible that calcium bound by 8-hydroxyquinolines can be more easily accessible for competition with *o*-CC and this could also contribute to the observed differences.

Summing up the above reported, one of the major findings of this study is the notice that some known chelators might affect negatively magnesium and/or calcium homeostasis in humans. This should be taken in the consideration when preparing novel chelators for potential clinical use. This situation is well known for EDTA. Although this compound has high affinity for metals, its non-selectivity represents an important disadvantage and obstacle for its larger clinical use. The fact that it can chelate strongly both calcium and magnesium ions was demonstrated in this study and previously as well [40]. Its chelation effect was employed therapeutically as EDTA has been tested for calcium removal from atherosclerotic vessels. However, long-term exposure to EDTA leads to side effects, which encompasses beyond renal toxicity, also overshoot hypocalcaemia [16, 41].

To demonstrate biological relevance of our data, the chelating potential was tested in four most effective chelators of calcium ions in the last step. Calcium is an important factor for physiological aggregation of platelets [42]. The hypothesis was that if the calcium chelation by our compounds is biologically relevant, it should stop the platelet aggregation in concentrations about 1 mM based on two facts: (1) the concentration of calcium in blood is about 2 mM, but during the experiment, the blood is diluted with the same volume of saline and (2) the selected chelators formed complexes with calcium in the stoichiometric ratio 1:1 (Fig. 8A–D) [43]. This method might appear at first sight unsuitable in many cases, as the tested compound can block platelet aggregation by other mechanism(s). Such potential confounders can be filtered out by additional testing. First of all, calcium is required for different aggregation pathways, so it is possible to use different inducer of platelet aggregation, if inhibition will be observed in lower concentration as it does correspond to the stoichiometry of the complex. We employed arachidonic acid, as we have large experiences with this inducer, but in principle another triggers (e.g., ADP, thrombin activating sequence, collagen) can be used as well. Moreover, even clinically used acetylsalicylic acid is not able to completely block platelet aggregation induced by arachidonic acid in whole blood [20] in contrast to chelation of calcium which can represent another proof of chelation. Other confounding aspect might be mediated by magnesium ions whose concentration in blood ranges between 0.75 and 0.95 mM [3]. This was, however, not an important obstacle in our experiments even if three from four tested compounds chelated magnesium as well: (1) the magnesium concentration in the testing cuvette was again ½ of that in blood due to abovementioned dilution of the sample and (2) there were no clear differences between the effect of EDTA and EGTA, although the latter is not able to chelate magnesium. Other metals, like iron and copper, are presented in its free form in negligible or low concentrations, and hence had apparently no effect on the assay, either.

In summary, this paper reported a preparation of a simple but precise competitive in vitro method for detection of calcium and magnesium chelation coupled with ex vivo confirmation of calcium chelation in platelet aggregation. There are no standard specific methods for assessment of calcium and magnesium chelation. Most researchers employed non-competitive methods. They either determined metal stability constants or carried out UV–Vis spectrophotometry frequently in combination with additional methods for confirmation of the obtained data and determination of chelating site (e.g., infrared spectroscopy, isothermal titration calorimetry, circular dichroism, mass spectrometry) [11, 39, 44–46]. Metal stability constants are a very suitable mean but require some knowledge and experiences, while analysis of UV–Vis spectra might not be simple in some situations as calcium and magnesium complexes might not absorb in visible area [45, 46], and hence the detection must be performed in UV area and this might be more challenging, i.e., due to interference with absorption of the solvent (e.g., DMSO) required for poorly water soluble compounds. Other mentioned methods require not only experiences, but also more expensive devices, and hence are not suitable for every laboratory. For this reason, we suppose that our method can be a good alternative to standard determination of metal stability constants as it does not require specific equipment and long-term experiences. Moreover, it has also some advantages including direct comparison among different compounds tested, suggestion of the complex stoichiometry in case of strong chelation and, last but not least, it enables detection of both calcium and magnesium chelation with one indicator. On the other hand, it has also some limitation, i.e., it cannot compare the activity between strong chelators with the same denticity.

## Conclusion

Following our research group’s previously published methodologies for the determination of metal ions, we have successfully developed a similar method for the determination of the degree of chelation of calcium and magnesium ions. The method has been transferred to 96-well microplates at pH 7.5 and has been successfully verified on 21 chemically diverse metal chelators encompassing a group of 8-hydroxyquinolines. This method is selective and cost-efficient, easy to perform, and rapid when using 96-well microplates. Furthermore, the biological relevance of the obtained data in relation to calcium can be verified using human platelet aggregation. As the second outcome, we have found that chelation activity of clinically used metal chelators toward calcium and magnesium was not negligible.

## Supplementary Information

Below is the link to the electronic supplementary material.

Supplementary file1 (PDF 737 kb)

## Data Availability

Research data for this paper are available at https://zenodo.org/uploads/13897072.
