# Supplementary material for: Rapid method for screening of both calcium and magnesium chelation with comparison of 21 known metal chelators
Source: J Biol Inorg Chem. 2024 Oct 18;29(7-8):785–800. doi: 10.1007/s00775-024-02078-6 (PMC11638374; doi:10.1007/s00775-024-02078-6)
Supplement: Supplementary file 1 — Supplementary file1 (PDF 737 kb) [file 775_2024_2078_MOESM1_ESM.pdf]

NOVEL SPECTROPHOTOMETRIC METHOD FOR SCREEING OF CALCIUM AND MAGNESIUM CHELATION WITH COMPARISON OF 21 KNOWN METAL CHELATORS

Lukáš Konečný[1], Zuzana Lomozová[2], Galina Karabanovich[3], Jaroslav Roh[3], Kateřina Vávrová[3] and Přemysl Mladěnka[1*]

[1] The Department of Pharmacology and Toxicology, Faculty of Pharmacy in Hradec Králové, Charles University, 50005 Hradec Králové, Czechia
[2] The Department of Pharmacognosy and Pharmaceutical Botany, Faculty of Pharmacy in Hradec Králové, Charles University, 50005 Hradec Králové, Czechia
[3] The Department of Organic and Bioorganic Chemistry, Faculty of Pharmacy in Hradec Králové, Charles University, 50005 Hradec Králové, Czechia

SUPPLEMENTARY DATA

5 pages

*correspondence

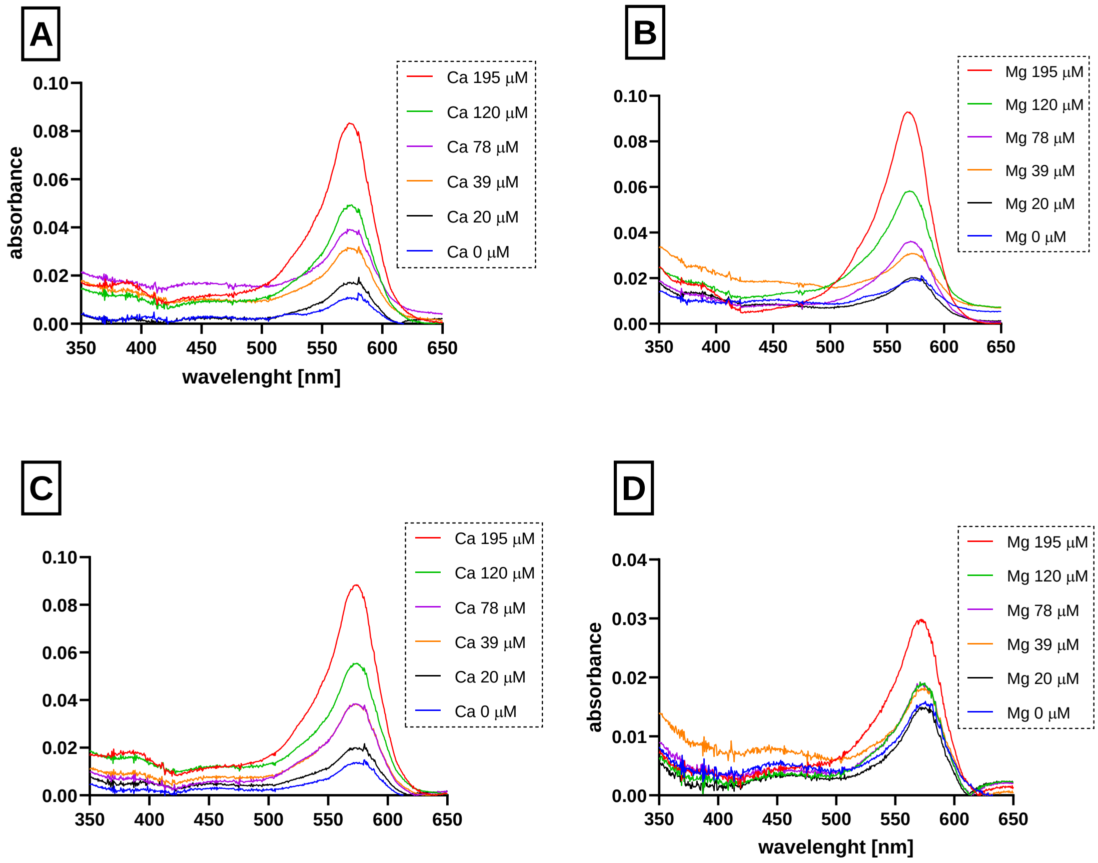

Supplementary figure 1. The spectra of indicator *o*-creslophtalein complexone (*o*-CC) dissolved in methanol and its complexes with calcium and magnesium ions. **A:** The complex of *o*-CC with calcium ions. **B:** The complex of *o*-CC with magnesium ions. **C:** The complex of *o*-CC with calcium ions after 15 minutes. **D:** The complex of o-CC with magnesium ions after 15 minutes. The final concentrations of both ions ranged from 0 to 195 μM whereas that of *o*-CC was 450 μM.

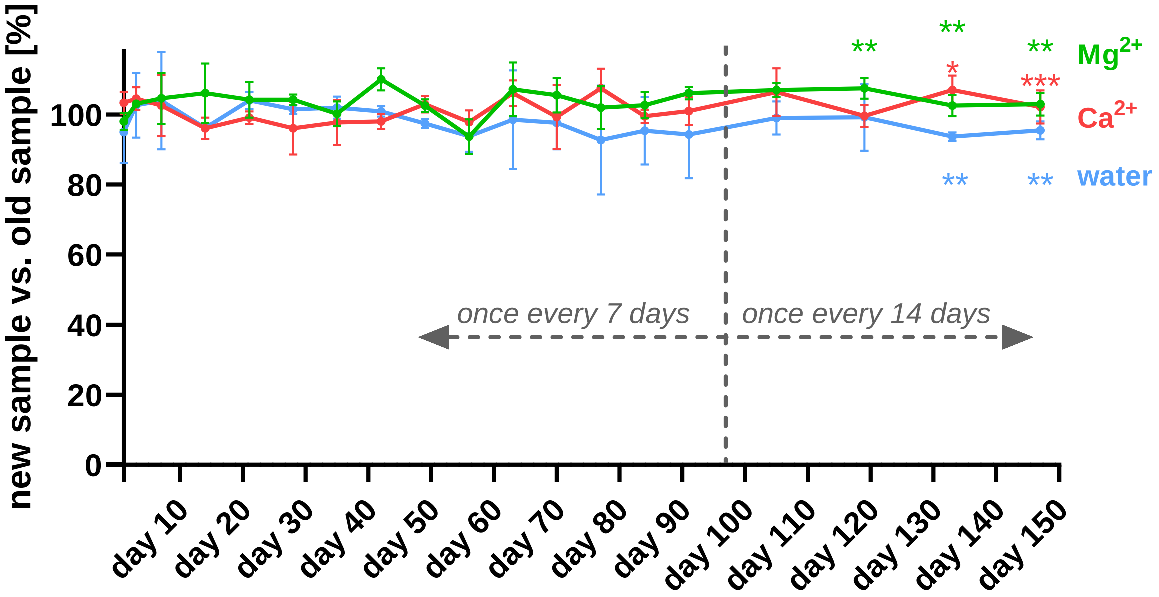

Supplementary figure 2. The stability of absorbance of the indicator *o*-creslophtalein complexone and its metal complexes. The absorbance ratio of the sample prepared on day 1 and the newly prepared sample is shown as a percentage. * p< 0.05; ** p< 0.01 *vs.* negative blank without metal ions. Absorbance was measured at 570 nm and compared to the fresh solutions prepared at the day of the measurement.

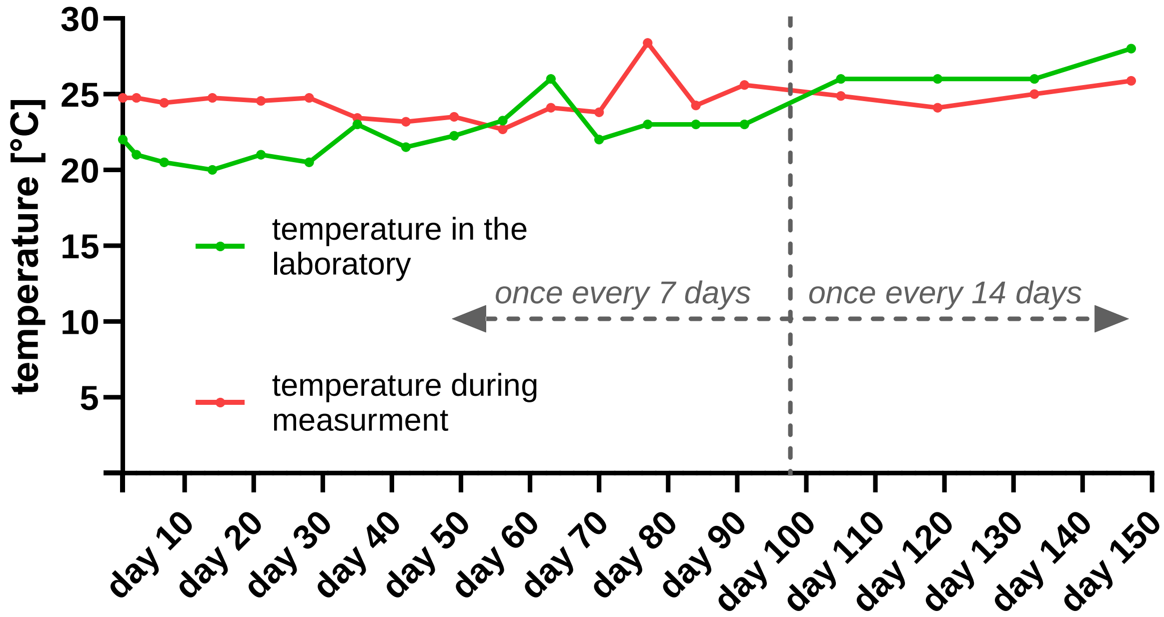

Supplementary figure 3. The temperature in the laboratory and during measurement.

Table 1. Correlation between absorbances and temperature.

	absorbance *vs.* temperature during measurement	
	slope (k)	*p*-value
water	-0,395	0,094
Ca + *o*-CC	-0,253	0,296
Mg + *o*-CC	-0,324	0,176

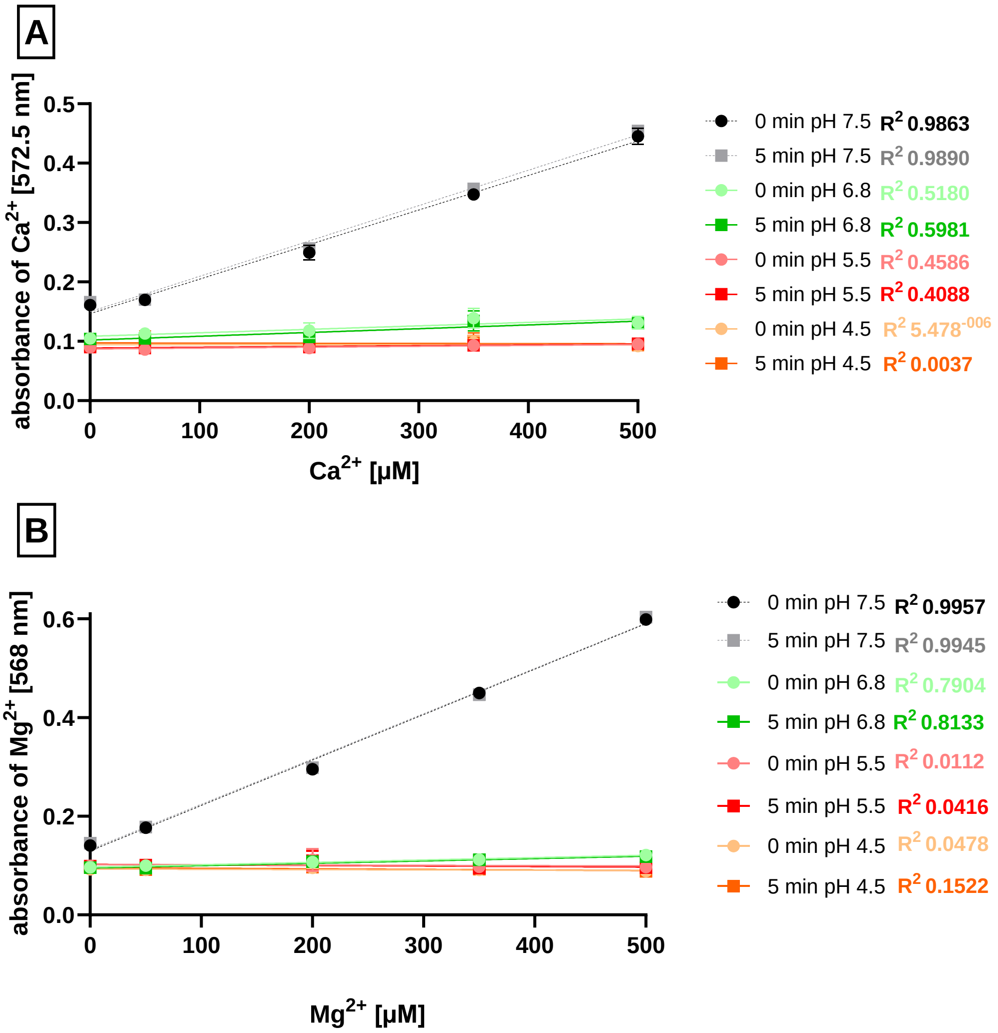

Supplementary figure 4. Absorbance of the mixtures of calcium and magnesium ions with the indicator *o*-creslophtalein complexone at pH 4.5, 5.5 and 6.8**.** The data for pH 7.5 are the same as from the main part of the article (Figure 5) and are shown solely for comparison.

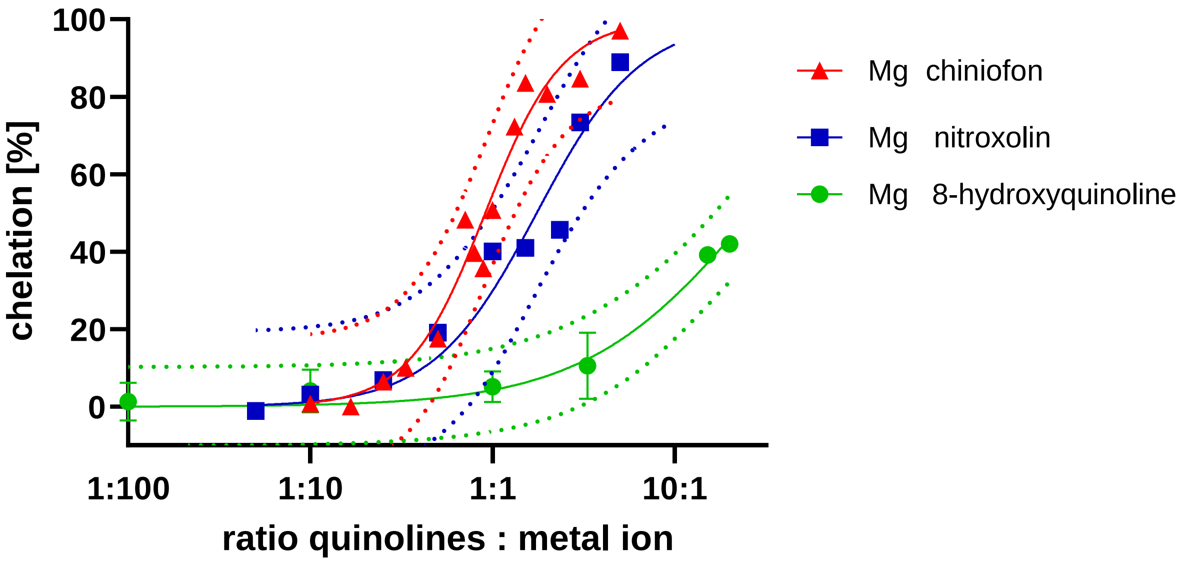

Supplementary figure 5. Comparison of the chelating activity of 8-hydroxyquinoline and two its more potent congeners toward magnesium ions. The magnesium chelating activity was measured at 568 nm. Results are presented as mean with 95 % confidence interval. The final concentration of magnesium ions was 0.5 mM while the final concentrations of tested compounds were in the range 5 nM to 10 mM depending on their chelating activity and solubility.
